# Isolation and Characterization of Outer Membrane Vesicles of *Pectobacterium brasiliense* 1692

**DOI:** 10.3390/microorganisms9091918

**Published:** 2021-09-09

**Authors:** Silindile Maphosa, Lucy Novungayo Moleleki

**Affiliations:** 1 Department of Biochemistry, Genetics and Microbiology, University of Pretoria, Lunnon Road, Pretoria 0028, South Africa; silindile.maphosa@up.ac.za; 2 Forestry and Agricultural Biotechnology Institute (FABI), University of Pretoria, Lunnon Road, Pretoria 0028, South Africa

**Keywords:** outer membrane vesicles (OMVs), *Pectobacterium brasiliense* 1692, virulence, competition

## Abstract

*Pectobacterium brasiliense* (Pbr) 1692 is an aggressive phytopathogen affecting a broad host range of crops and ornamental plants, including potatoes. Previous research on animal pathogens, and a few plant pathogens, revealed that Outer Membrane Vesicles (OMVs) are part of Gram-negative bacteria’s (GNB) adaptive toolkit. For this reason, OMV production and subsequent release from bacteria is a conserved process. Therefore, we hypothesized that OMVs might transport proteins that play a critical role in causing soft rot disease and in the survival and fitness of Pbr1692. Here, we show that the potato pathogen, Pbr1692, releases OMVs of various morphologies in Luria Bertani media at 31 °C. Scanning Electron Microscopy (SEM) and Transmission Electron Microscopy (TEM) confirmed the production of OMVs by Pbr1692 cells. Transmission Electron Microscopy showed that these exist as chain-, single-, and double-membrane morphologies. Mass spectrometry followed by Gene Ontology, Clusters of Orthologous Groups, Virulence Factor, CAZymes, Antibiotic Resistance Ontology, and Bastion6 T6SE annotations identified 129 OMV-associated proteins with diverse annotated roles, including antibiotic stress response, virulence, and competition. Pbr1692 OMVs contributed to virulence in potato tubers and elicited a hypersensitive response in *Nicotiana benthamiana* leaves. Furthermore, Pbr1692 OMVs demonstrated antibacterial activity against *Dickeya dadantii*.

## 1. Introduction

Soft Rot *Pectobacteriaceae* (SRP) are Gram-negative bacteria (GNB) responsible for annual crop losses amounting to millions of dollars worldwide [1]. *Dickeya* and *Pectobacterium* spp. are the primary soft rot pathogens causing wilting, blackleg, and tuber decay of potatoes (*Solanum tuberosum* L.), other vegetables, and ornamental plants [2,3]. First reported in Brazil, Pbr is not only found in different parts of the world but has also become a significant threat to the production of economically important crops globally [4,5,6]. Pbr, like other soft rot bacteria, exhibits many determinants that ensure their persistence in the environment and expansion into new territory and hosts. Similar to other SRP, the main virulence determinants are consignments of plant-cell-wall-degrading enzymes (PCWDEs), such as pectinases, cellulases, and proteinases, which make the genus effective in macerating plant tissue [1]. Apart from PCWDEs secreted via the Type 2 Secretion System (T2SS), SRP have an arsenal of virulence factors that elevate pathogenesis. These include iron acquisition, quorum sensing, fimbriae, flagella and motility, polysaccharides, and bacterial secretion systems (T1-, T3-, T4-, T5-, and T6SS), with outer membrane vesicles (OMVs) considered a bacterial Type 0 secretion system [1,7,8,9,10,11]. Indeed, the role of secretion systems in the coordinated interaction of bacteria with their environment, target hosts, and other bacteria is pivotal [12]. These systems release many proteinaceous and chemical substances for virulence and persistence from the cell cytosol to the extracellular milieu, neighboring cells, and host cells. Extensive studies of T1SS–T6SSs have determined their composition and the substances they deliver [13].

In general, Membrane Vesicles (MVs) form in GNB by pinching off the outer membranes (OMs), resulting in OMVs and Gram-Positive Bacteria (GPB) by disrupting the peptidoglycan layer forming microvesicles [14]. Owing to their essential role in transferring bioactive molecules from donor to recipient cells, MVs, particularly OMVs in GNB, are ubiquitous and conserved [7,10,11]. Although models of the mechanisms of biogenesis, cargo, and functions of OMVs continue to be reported, not much is known about the mechanism of loading and sorting of the cargo into the vesicles for transportation [15,16]. Outer membrane-derived OMVs are enriched in periplasmic proteins, while outer-inner membrane vesicles (OIMVs) have inner and outer membranes enriched in addition to periplasmic proteins and cytoplasmic contents. More so, Explosive OMVs (EOMVs) carrying cytoplasmic proteins in addition to periplasmic proteins have been reported [16,17,18]. Studies also report OMVs of larger sizes, aggregates, chains, and tubes [19]. However, there are no baselines for isolation methods; hence, the types, cargo, and specific functions of OMVs will vary from one species to another, making comparisons difficult [20,21]. We have yet to learn more about the species and strain-specific roles of OMVs of phytopathogenic bacteria in bacteria–bacteria and bacteria–host interactions, as well as their potential to control plant diseases.

So far, we know that OMVs have physiological, protective, and adaptative roles in the environment, as reviewed in [22,23]. Phytopathogenic bacteria produce OMVs involved in colonization, virulence, host immune elicitation or evasion, defense neutralization, and biofilm formation [24,25,26]. Therefore, bacterial cells communicate with each other and the environment through OMVs. This form of communication is without energy expenditure to transfer bacterial messenger molecules, toxins, and nutrient acquisition/scavenging systems that consequently affect the environment even though cargo sorting into and producing OMVs is likely resource depleting [14,15]. Moreover, the rapid release of toxins and misfolded proteins produced under stressful conditions, surface binding and antimicrobial agents, and hydrolytic enzymes ensures the survival of the producing bacteria [19]. Unlike other secretion mechanisms that solely transport soluble material, OMVs enable bacteria to secrete insoluble molecules complexed with soluble material [27]. Since they transport a set of biological macromolecules typifying most secretion systems’ substrate, OMVs play a critical role as the GNB secretion systems’ succor system. Therefore, bacterial OMVs provide relief from the secretion burden to delivery systems [7]. OMVs ferry lipopolysaccharides (LPS), phospholipids, peptidoglycan, Outer Membrane Proteins (OMPs), cytoplasmic proteins, nucleic acids, ion metabolites, and signaling molecules as part of their structure and in their lumen for various destination outcomes [28,29]. External surface association of toxins and nucleic acids with OMVs also exists [30]. Upon reaching the targeted destination, OMVs release their cargo, which may be involved in different biological roles [22,31]. Exploration of OMV cargo in other GNB points to several such roles, including increased virulence, immune activation or suppression, biofilm formation, inter- or intraspecies and interkingdom delivery of defensive and offensive molecules, respectively, uptake, and pathogen adherence or detachment [24,32,33,34]. For example, Enterotoxigenic *Escherichia coli* (ETEC) vesicles are essential in disseminating heat-labile enterotoxin to host cells during bacterial infection leading to diarrhoea [35]. The OMV-enriched proteome of two human microbiota, *Bacteroides fragilis* and *Bacteroides thetaiotaomicron* showed high glycosidases and proteases, including those active in vitro [36]. Moreover, proteomics has identified different putative biologically active molecules like nucleotides and immunogenic peptidoglycan [37]. OMV proteins are involved in communication, nutrient acquisition, stress responses, and virulence [23]. Some of the identified OMV-specific proteins in previous studies are uncharacterized hypothetical proteins; hence, it may be challenging to predict functions for these or fully understand the proteins found in the vesicles [38].

Pbr1692 is more virulent than other *Pectobacterium* spp. and is able to outcompete other members of SRP under different environmental conditions [39,40]. We presupposed that OMVs could contribute to the delivery of PCWDEs, toxins, and effectors involved in either virulence or competition. In this work, we established that Pbr1692 naturally releases MVs throughout its growth. These OMVs could macerate potato tuber tissue, elicit a hypersensitive response (HR) on *Nicotiana benthamiana* leaves, and were able to inhibit the growth of another SRP, *D. dadantii*. We carried out proteomics analysis of isolated vesicles to identify the OMV-associated proteins. Functional annotations showed that diverse functions could be ascribed to Pbr1692 OMVs centralized in virulence and fitness.

## 2. Materials and Methods

### 2.1. The Strains and Growth Conditions

Pbr1692 and *D. dadantii* (with plasmid pMP7605 conferring gentamycin resistance) were cultured in Luria Bertani (LB) media at 31 and 32 °C, respectively, with shaking at 120 rpm for 12–14 h. Where required, for the growth of *D. dadantii*, growth media were supplemented with 15 µg/mL gentamycin antibiotic (Sigma-Aldrich, St. Louis, MO, USA).

### 2.2. Scanning Electron Microscopy Analysis of Cells

Five milliliters of Pbr1692 cells in exponential and stationary phase were pelleted at 3000 rpm for 3 min. The supernatant was discarded, and then cell pellets were washed with 0.1 M phosphate buffer pH 7.4 on a rotator and after the fixation and post-fixation steps thrice for 10 min. First, the cells were resuspended in 2.5% glutaraldehyde fixative for 30 min, post-fixed with 1% osmium tetroxide (*w*/*v*) for 30 min, and then dehydrated by a graded series of ethanol (30%, 50%, 70%, and 90%) for 10 min. The cells were then dehydrated by 100% ethanol twice for 10 min and once for 40 min. Next, the cells were pelleted and embedded in hexamethyldisilazane (HMDS): ethanol mixture for 1 h twice. Following this, fresh HDMS was added, and then the cells were left to dry. The cells were then mounted and coated with carbon for visualization using the Zeiss Crossbeam 540 Field Emission-Scanning Electron Microscope (FE-SEM) (Zeiss, Oberkochen, Germany) operated at 1.00 kV.

### 2.3. Transmission Electron Microscopy Analysis of Cells

Five milliliters of Pbr1692 culture in the stationary phase were pelleted at 3000 rpm for 3 min. The supernatant was discarded, and then the cell pellet was washed with phosphate buffer on a rotator and after the fixation and post-fixation steps thrice for 10 min each time. The cells were resuspended in 2.5% glutaraldehyde fixative for 30 min and then post-fixed with 1% osmium tetroxide (*w*/*v*) for 30 min. After that, a graded series of ethanol (30%, 50%, 70%, and 90%) was used to dehydrate the cells for 10 min. Dehydration by 100% ethanol was done twice for 10 min and once for 40 min. Dehydrated cells were mixed with epoxy resin:ethanol mixture for 1 h twice. Fresh epoxy was added, and then the cells were dried in an oven for over 24 h for polymerization. The embedded sample was trimmed, sectioned, and stained for visualization using the transmission electron microscope (TEM) (JOEL JEM 2100F, JOEL Ltd., Tokyo, Japan).

### 2.4. Isolation of OMVs

OMVs were isolated at the stationary growth phase of Pbr1692 (OD_600_ of ~1–1.2) according to [41,42] with modifications. First, the Pbr1692 culture was subjected to low-speed centrifugation at 16,000× *g* for 20 min at 4 °C to pellet the cells. After that, cell debris and macromolecules were removed by high-speed centrifugation at 38,000× *g* for 1 h at 4 °C. The cell-free supernatant was filtered using a 0.22 µm bottle filter top under vacuum to remove residual cells (Merck, Darmstadt, Germany). LB agar plates were inoculated with 200 µL of cell-free filtrates and incubated at 31 °C for 48 h to ensure total removal of bacterial cells. The cell-free supernatants were reduced 100-fold by concentration in Amicon^®^ Ultra-15 (MWCO 30 kDa) centrifugal units (Merck, Darmstadt, Germany). OMVs were pelleted from the concentrated supernatant in an SW 55 Ti Rotor (Beckman Coulter, Brea, CA, USA) at 145,000× *g* at 4 °C for 6 h and then resuspended in phosphate-buffered saline (PBS) (Sigma-Aldrich, St. Louis, MO, USA). OMVs for virulence assays were washed twice in the SW 55 Ti Rotor at 145,000× *g* at 4 °C for 6 h. Protein concentration was checked using the nanodrop at A_280_. OMV samples were stored at −20 or −80 °C after freezing in liquid nitrogen.

### 2.5. Negative Staining of OMVs

Purified OMVs were spotted on carbon-coated grids for adsorption for 5 min. The vesicles were, after that, negatively stained with 1% (*w*/*v*) uranyl acetate for 3 min. Finally, the OMVs were visualized using TEM (JOEL JEM 2100F, JOEL Ltd., Tokyo, Japan).

### 2.6. Nanoparticle Tracking Analysis and Bradford Assay of Cell-Free Supernatants

Isolated OMV samples and Pbr1692 cell-free supernatants were analyzed using the NanoSight NTA v3.3 (Malvern Panalytical, Malvern, UK) to determine nanoparticle concentration and distribution. Cell-free supernatants were prepared for NTA according to [25] with slight modifications. Three biological replicates of Pbr1692 cells were grown to the exponential (OD_600_ of ~0.5) and stationary growth phase (OD_600_ of ~1.0). Cells were pelleted out of culture using low-speed centrifugation at 16,000× *g* for 20 min twice at 4 °C. The cell-free supernatants were filtered under vacuum to remove residual bacterial cells. A test volume of 1.5 mL of each replicate was injected into the NTA and analyzed in triplicate (each run = 30 s video). The videos were captured and processed using NTA 3.3 Dev Build 3.3.104 (Malvern, Panalytical, Malvern, UK). In between runs, samples were advanced to introduce fresh sample aliquots for quantification. The camera sensitivity and detection threshold were optimized per video, and the temperature was set between 20 and 22 °C. Readings were analyzed using the NTA software version 3.3. LB media were used as a blank. The Bradford reagent was used to determine the protein concentration of the cell-free supernatants (Bio-Rad Laboratories, Hercules, CA, USA ) using bovine serum albumin (BSA) as a standard (Sigma-Aldrich, St. Louis, MO, USA).

### 2.7. Protein Extraction and Digestion

OMV proteins of three biological replicates were extracted according to the BGI Tech Solutions Co., Ltd. (Hong Kong, China) protocol. The OMVs were mixed with 1 mL PBS and centrifuged at 1000× *g* at 4 °C twice for 1 min. Each time, the supernatant was discarded. Two steel beads, a 1X cocktail with appropriate amounts of SDS L3 and EDTA were added, and then the tube was incubated on ice for 5 min. After incubation, 10 mM dithiothreitol (DTT) was added. The mixture was placed on a grinder at 60 Hz frequency for 2 min to crush the tissue and then centrifuged at 25,000× *g* at 4 °C for 15 min. The supernatant was transferred into a fresh tube, and 10 mM DTT was added before incubation in a water bath at 56 °C for 1 h. Next, iodoacetamide (IAM) at a final concentration of 55 Mm was added, and then the samples were placed in a dark room for 45 min. Next, cold acetone was added to the protein solution at a ratio of 1:5, and then the samples were refrigerated at −20 °C for 30 min. After freezing, the samples were centrifuged at 25,000× *g* at 4 °C for 15 min, and then the supernatant was discarded. The protein was precipitated by air-drying before the addition of lysis buffer without SDS L3. The grinder at 60 Hz (2 min) was used to promote protein solubilization. Post solubilization, the samples were centrifuged for 15 min at 25,000× *g* at 4 °C to recover the protein-containing supernatant.

Proteins were separated by SDS PAGE gel electrophoresis and extracted from the gel strips. The strips were decolorized in ammonium bicarbonate (NH_4_HCO_3_):acetonitrile (ACN) mixture (*v*/*v*) at 37 °C for 30 min, then centrifuged and dried. The gels were dehydrated using 500 µL acetonitrile twice and then left to air dry. The gels were covered in 10 mM DTT and incubated at 56 °C for 1 h. After that, the gel was soaked with 55 mM IAM and kept in the darkroom at room temperature for 45 min. The samples were washed with discolorizing solution and then with pure water before adding 500 µL acetonitrile. The samples were vortexed for 5 min and, after the addition of acetonitrile, were left to dry thoroughly. The dry gel samples were digested by covering them in enzyme solution diluted to 0.01 µg/µL with 25 mM NH_4_HCO_3_ for 30 min at 4 °C and then incubating in buffer overnight at 37 °C. Fifty percent and 100% acetonitrile were added in series before centrifugation at 5000× *g* each time before the samples were freeze-dried.

### 2.8. High-Performance Liquid Chromatography (HPLC)

The freeze-dried peptide samples were reconstituted with mobile phase A (2% ACN, 0.1% FA), centrifuged at 20,000× *g* for 10 min. The supernatant was taken for injection and separated by a Shimadzu LC-20AD model nanoliter liquid chromatograph. The sample was first enriched in the trap column and desalted and then channeled into a tandem self-packed C18 column (75 μm internal diameter, 3 μm column size, 15 cm column length). Separation was at a flow rate of 300 nL/min by the following effective gradient: 0–6 min, 6% mobile phase B (98% ACN, 0.1% FA); 6–40 min, mobile phase B linearly increased from 6% to 25%; 40–48 min, mobile phase B rose from 25% to 40%; 48–51 min, mobile phase B rose from 40% to 90%; 51–55 min, 90% mobile phase B; 55.5–60 min, 6% mobile phase B. The nanoliter liquid phase separation end was directly connected to the mass spectrometer.

### 2.9. Electrospray Ionization Mass Spectrometry (ESI-MS/MS)

Liquid phase chromatography-separated peptides were passed to the ESI tandem mass spectrophotometer, TripleTOF 5600 (SCIEX, Framingham, MA, USA). Nanospray was used as an ion source (SCIEX, Framingham, MA, USA), and the emitter was a needle (New Objectives, Woburn, MA, USA) drawn from quartz material. The specific applied parameters of ion spray voltage 2300 V, nitrogen pressure 35 psi, spray gas 15, and spray interface temperature 150 °C were set. Scanning in high sensitivity mode, the cumulative scan time was 250 ms, and the scan quality range was between 350 and 1500 Da. Based on the MSI scanning information and the ionic strength in the MSI spectrum from high to low, the first 30 ions that exceeded 150 cp were fragmented, and the MS2 information was scanned. The screening was done according to the following criteria: (a) the *m*/*z* range was 350–1250 *m*/*z*; (b) the number of charges was 2–5 charges; (c) the dynamic elimination of the parent ion was set to half of the peak time (~12 s); and the fragmentation of the same parent ion did not exceed 2 times. The scan accumulation time MS2 mass spectrum was 50 ms. The collision energy was set to ‘Rolling Collision Energy’.

### 2.10. Database Search and Quantification

The MS/MS experimental data were aligned with theoretical MS/MS data from a Pbr database (14,096 sequences). First, the MS/MS raw data were converted to an *mgf file and searched for matches in the database using Mascot software (Matrix Science, London, UK; version 2.3.02). A fragment mass tolerance of 0.1 Da and a peptide tolerance of 0.05 Da were set. Carbamidomethylation cysteine (C) was the fixed modification. The oxidation of methionine (M), conversion of N-terminal glutamine (Gln) to pyroglutamic acid (pyro-Glu), and the deamination of N-terminal glutamine (Q) were set as variable modifications. Trypsin specificity was set to allow 1 missed cleavage. The search results were rescored using Percolator to improve the matching accuracy. Then, the output was filtered by a 1% false determination rate (FDR) at the spectral level (PSM-level FDR ≤ 0.01) to obtain the significant identified spectrum and peptide list. Proteins were linked to peptides, and a series of protein groups were generated. The intensity-based absolute protein quantification (iBAQ) was carried out as previously described [43].

### 2.11. Sequence Analysis and Annotations

The protein sequences were first retrieved from the UniProt database to establish their functional annotations. The Gene Ontology (GO) term claims for the proteins were assigned using the GO international standard gene function classification system [44]. The GO functional annotation included protein2go and go2protein. The subcellular localization of each identified protein was predicted using PSORTb v3.0.3 [45]. Protein classification was carried out using the Clusters of Orthologous Groups of Proteins System (COG) [46]. Virulence proteins were identified using the Virulence Factor DataBase (VFDB) [47]. Carbohydrate-Active enZYme (CAZyme) annotation was performed using dbCAN2 [48]. We also used a Comprehensive Antibiotic Resistance Database (CARD) to identify proteins essential for the persistence of Pbr1692 in the presence of antibiotics [49]. Type 6 secreted effectors (T6SE) were predicted using Bastion6 [50].

### 2.12. Virulence Assays of Pbr1692 OMVs

#### 2.12.1. Protease Activity of OMVs

Twenty micrograms of OMVs was mixed with loading buffer (without β-mercaptoethanol) and incubated at room temperature for 10 min. The samples were then loaded onto a 12% SDS-0.1% gelatin-polyacrylamide gel (*v*/*v*). The gel was run at 150 V (30–50 mA) using a 1X electrophoresis buffer. Gelatinase activation was performed by washing the gel three times with gelatinase renaturation buffer for 20 min each time with agitation. After that, the gel was incubated in a washing buffer for 24 h at 37 °C. For staining, the gel was incubated in Coomassie Brilliant blue G 250 gel staining solution for 1 h at room temperature in a shaker. The gel was destained in the destaining solution until visible clearing was observed.

#### 2.12.2. Maceration of Potato Tubers

Tap-water-washed *Solanum tuberosum* L. (cv. Mondial, a susceptible cultivar) potato tubers were surface sterilized by soaking in 10% (*v*/*v*) sodium hypochlorite (NaOCl) for 10 min. The potatoes were rinsed three times with sterile distilled water and then once with 96% ethanol. The potatoes were then allowed to air dry before 1 cm holes were punched with a sterile tip. The generated wounds were inoculated with 10 μL OMV suspension in PBS (~1 × 10^11^ OMVs/mL), OD_600_ of 1 Pbr1692 cells, and Pbr1692 cell-free supernatant. For the negative control, sterile PBS was inoculated. The inoculation sites were then sealed with petroleum jelly, and the potatoes were incubated under humid conditions at 31 °C for 72 h. The lesion sizes were measured at this time. The experiment was carried out using three biological replicates, two independent times.

#### 2.12.3. *Nicotiana benthamiana* Elicitation of Hypersensitive Response by Pbr1692 OMVs

Two two-week-old *N. benthamiana* plants were infiltrated by piercing three different leaves per plant with a needle and saturating each leaf with 1 mg/mL OMVs, cell-free supernatant, and OMV-wash supernatant as a control through the puncture two independent times.

### 2.13. Antimicrobial Activity of OMVs

Frozen stocks of target soft rot bacterium, *D. dadantii* (harboring pMP7605 which confers gentamycin resistance), and Pbr1692 were streaked on LB agar plates and incubated overnight at 32 and 31 °C, respectively. Single colonies were picked from the plates to inoculate 20 mL of fresh LB broth and incubated as above with shaking at 120 rpm for 12–14 h. Overnight cultures were then normalized to OD_600_ of 0.5. Twenty microliters of OMV suspension in PBS (~1 × 10^11^ OMVs/mL) and *D. dadantii* (OD_600_ of 0.5) was mixed in a 1:1 ratio and spotted on an LB agar plate and then incubated at 32 °C for 16 h. A volume of 20 µL *D. dadantii* (OD_600_ of 0.5) cells was also mixed with an equal volume of the normalized Pbr1692 cells (OD_600_ of 0.5) as a positive control. PBS was used as a negative control. All the spots were scrapped into 1 mL LB post-incubation, serially diluted with sterile water, and then plated on LB plates supplemented with gentamycin. The single colonies were counted to enumerate viable cells in three independent plates.

### 2.14. Statistical Analysis

In this study, experiments were performed in triplicate two independent times. Where applicable, a student t-test and analysis of variance using R version 3.6.1 were performed to determine statistical significance, and a *p*-value less than 0.05 or 0.01 (*p* < 0.05 or *p* < 0.01) was a statistically significant difference.

## 3. Results

### 3.1. Identification of Pbr1692 OMVs

**Figure 1:**
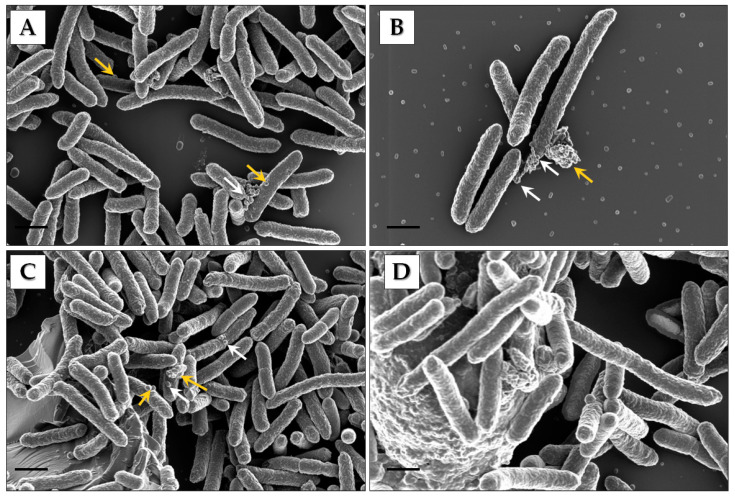
Identification of OMVs produced by Pbr1692. (**A**,**B**) In the SEM images, gold arrows indicate OMVs of cells and OMV aggregates in the exponential growth phase. The white arrows show vesiculation “hot spots”. (**C**) The image of cells in stationary growth shows OMVs at the surface of the bacteria indicated by white arrows. Gold arrows indicate OMV aggregates and “hot spots”. (**D**) The image shows OMVs embedded in the biofilm matrix-like backbone. Scale bars = 1 µm.

To establish whether Pbr1692 produces OMVs in rich media, SEM was used to analyze cells cultured to exponential and stationary growth phases (Figure 1A–D). OMVs were observed budding off from or on the surfaces of rod-shaped bacterial cells both in the exponential and stationary phases. OMV aggregates and biofilm-like matrix structures were observed at both time points (Figure 1B,D). In addition, there were distinct areas on the bacterium where OMV clustering signaled where production was concentrated, “hot spots” (white arrows).

### 3.2. Electron Microscopy and Nanoparticle Analysis of Vesicles

**Figure 2:**
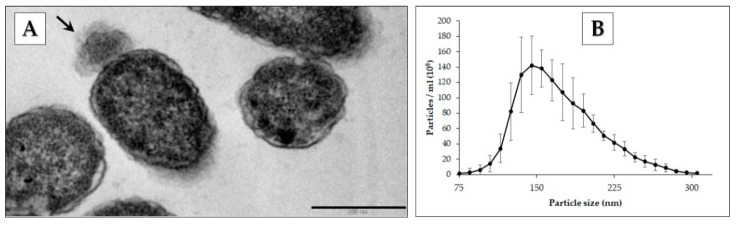
TEM identification of budding vesicles on Pbr1692 cells and isolated OMV size and distribution. (**A**) The arrow indicates an OMV budding from a Pbr1692 cell. Bar: 200 nm. (**B**) NTA size distribution and quantification of isolated OMV assessment shows the distribution of Pbr1692 OMVs with an average size of 173.7 ± 1.9 nm isolated in the stationary growth phase.

Pbr1692-pelleted cells were analyzed using TEM to observe budding vesicles (Figure 2A). The cell density and total protein concentration of exponential and stationary cell-free supernatants were OD_600_ of 0.547 and 1.050 with a total protein of 60 and 102 µg/mL, respectively. However, with no statistically significant difference in nanoparticle concentration or size distribution (Figure S1), OMVs were isolated in the stationary phase and analyzed using NTA. The OMV particle size distribution of 75–355 nm is shown in Figure 2B. The recorded average size was 173 ± 1.9 nm, and the mode diameter was 150.5 ± 7.9 nm.

**Figure 3:**
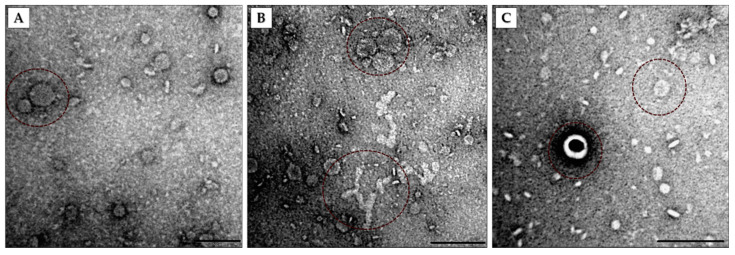
Transmission electron microscopy of OMVs isolated from Pbr1692 in the stationary growth phase. (**A**) The TEM image shows OMVs of different sizes. (**B**) The image shows OMV aggregates (top) and the OMV chain-like morphology (bottom). (**C**) The dotted circles indicate a single-membrane vesicle and a double-membrane OIMV vesicle with a halo around. Scale bars = 200 nm.

The isolated OMVs were negatively stained and analyzed using TEM (Figure 3). Vesicles of different sizes and morphologies were observed. OMVs of varying sizes are shown (Figure 3A). Chain-like and aggregated OMVs were also identified (Figure 3B). Some of the vesicles had double membranes (Figure 3C).

### 3.3. Proteomic Analysis of OMVs

Pbr1692 encodes 4135 proteins in its genome (https://www.uniprot.org/proteomes/UP000464068 accessed on 15 June 2021). The TripleTOF 5600 mass spectrophotometer was used to analyze the digested proteins of OMV samples isolated from three independent cultures to produce 119,240 spectra in total. Mascot analysis of the mass spectrometry generated data by searching against a Pbr constructed database was used to identify the OMV proteins. A total of 451 proteins were identified as potential OMV cargo of Pbr1692 in OMV-enriched fractions of three independent culture supernatants (Table 1).

**Figure 4:**
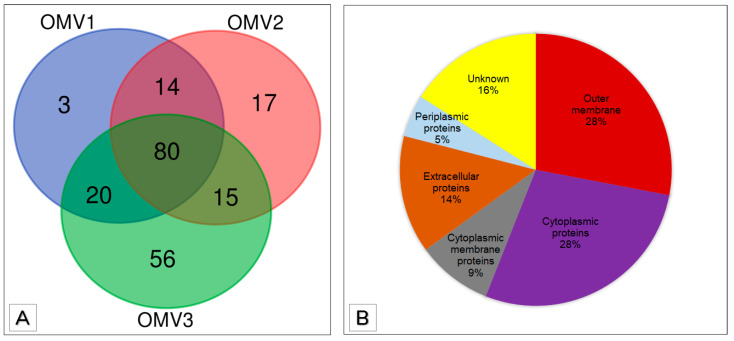
Shared and exclusive OMV-associated proteins of biological replicates and shared protein localization predictions. (**A**) The Venn diagram indicates shared and exclusive proteins of the three biological replicate OMVs isolated from Pbr1692 cultures. Eighty proteins were found present in all three biological replicates. (**B**) The pie chart shows the percentages of shared OMV proteins’ predicted localizations.

A cut-off of two or more predicted peptides per identified protein was used to filter the proteins identified in the three biological replicates shown in Table 1. In total, 117, 126, and 171 proteins were considered OMV cargo of OMV1, OMV2, and OMV3, respectively (Figure 4). A total of 114 (97%) of the 117 proteins identified in OMV1 were present in the other two replicates (Figure 4). Of the 126 proteins identified in the sample OMV2, 109 (87%) were present in the other two replicates. Lastly, among OMV3 proteins, 115 (67%) placed in replicate OMV1 and OMV2. OMV1, OMV2 and, OMV3 had 3, 17, and 56 unique proteins, respectively (Figure 4A). A total of 129 proteins present in at least two biological replicates were taken for further analysis as Pbr1692 OMV-associated proteins and cargo (Table S1). The biological replicate protein numbers suggest a high degree of consistency and reproducibility. In total, 80 (62%) of the 129 proteins were present in all three replicates, and 49 (38%) were present in two biological replicates. To predict the subcellular localization of the OMV proteins, PSORTb v3.0.3 was employed. Most of the OMV proteins were outer membrane (28%) and cytoplasmic proteins (28%) (Figure 4B). Other locale predictions included cytoplasmic membrane proteins (9%), extracellular proteins (14%), and periplasmic proteins (5%). Subcellular predictions of 16% of the OMV proteins were not assigned subcellular localizations.

The iBAQ was used to establish the most abundant proteins in OMVs. The top 50 most abundant proteins are shown in Table 2. Among the most abundant proteins, most were outer membrane proteins. Very few proteins in the top 50 were identified as cytoplasmic membrane (3) and periplasmic (1) proteins. Nine proteins in the most abundant OMV proteins had no localization information, according to the PSORTb tool. Gene ontologies were also assigned to the most abundant proteins (Table 2).

### 3.4. Functional Annotation of OMV Proteins

Gene Ontology (GO), Clusters of Orthologous Groups (COG), Carbohydrate Active enZYme (CAZyme), Virulence Factor DataBase (VFDB) integrated with VFanalyzer, Antibiotic Resistance Ontology (ARO), and Bastion6 T6SE annotations were employed to describe the potential functions of the OMV proteins. Proteins were annotated in GO terms to comprehensively provide all possible claims of their properties within the following ontologies: molecular, cellular, and biological process functions. In total, 240 GO annotations were assigned to 104 OMV proteins. The WEGO output (https://wego.genomics.cn/ accessed on 26 April 2021) was used to visualize the respective ontology entries of the proteins with assigned GO terms within the three ontologies and the number of proteins associated with each group. A total of 81 biological functions and 80 cellular component functions were assigned. Under the ontology of biological processes, the highest number of proteins was found in annotated cellular (GO:0009987) and metabolic process (GO:0008152) functions. The cellular component terms cell (GO:0005623) and cell part (GO:0044464) represented 59.6% and 58.7%, respectively.

**Figure 5:**
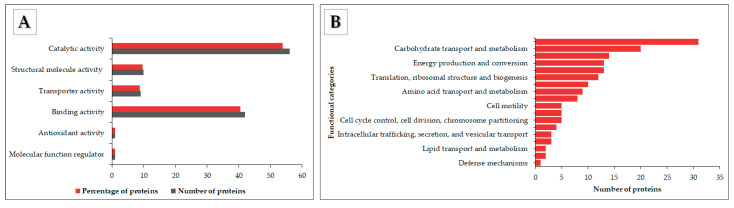
OMV protein molecular functions and protein COG classification. (**A**). The graph shows the molecular ontology entries against the number and percentage of associated proteins. (**B**) The chart shows protein functional group categories and the corresponding number of proteins associated with each cluster.

A total of 79 molecular function GO annotations were assigned (Figure 5A). In total, 56 (53.8%) of the 104 proteins were assigned catalytic activity functions (GO:0003824). The most abundant in this entry had hydrolase (GO:0016787), transferase (GO:0016740), and lyase (GO:0016829) activity. Proteins exhibiting phospholipase A1 or A2 activity, polygalacturonase activity, peptidase activity, and cellulase activity were among the identified hydrolases. Nine proteins (8.7%), six (5.8%), and three (2.9%) were annotated oxidoreductase activity, catalytic activity acting on a protein, and peptidoglycan muralytic activity, respectively. Forty-two proteins exhibiting binding ability (GO:0005488) made 40.2% of the total number of annotated proteins. Seven (6.7%) proteins were annotated drug binding activity. Other entries in the molecular ontology included structural molecule activity (GO:0005198), transporter activity (GO:0005215), a molecular function regulator role (GO:0098772), and antioxidant activity (GO:0016209). Sets of COGs established more useful classification clues (Figure 5B). COGs linked the proteins to an evolutionary trail to clarify the proteins’ roles. The cluster with the highest number of proteins included cell wall/membrane/envelope biogenesis groups. Relatively high numbers were also assigned to groups with carbohydrate transport and metabolism, energy production and conversions, and inorganic ion transport and metabolism functions. COG analysis predicted generalized positions for eight proteins and did not establish cluster links for 13 proteins. The other protein cluster groups identified are shown in Figure 5B.

### 3.5. Identification of OMV Virulence Factors, Carbohydrate-Active Proteins, Antibiotic Agents, and T6SEs

**Figure 6:**
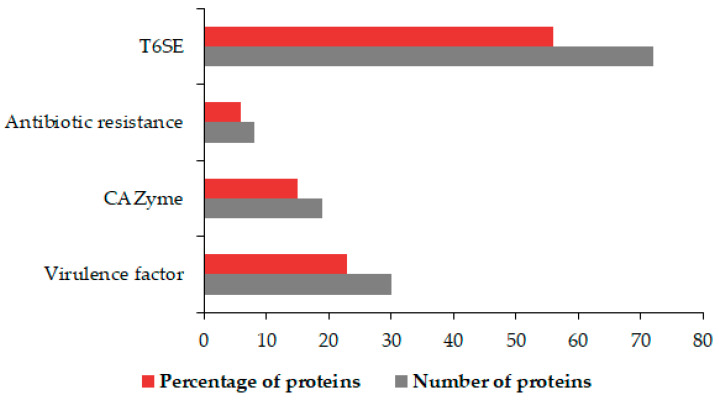
OMV proteins associated with virulence, carbohydrate metabolism, stress resistance, and competition. The graph shows the number and percentages of OMV virulence factors, CAZymes, antibiotic resistance agents, and T6SE.

We established further which OMV proteins exhibit virulence, carbohydrate-active, antibiotic resistance, and antibiotic properties (Figure 6).

Thirty-one virulence proteins were identified using the Virulence Factor DataBase (VFDB). The identified Virulence Factors (VF) included six pectate lyases with carbohydrate metabolism roles, attachment invasion locus protein, adhesin, and ATPase activity. Other VF proteins found had catalase (tr|A0A0M2F307|A0A0M2F307_9GAMM), esterase (EstA) (tr|A0A0M2F3J1|A0A0M2F3J1_9GAMM), and contact-dependent inhibition A (CdiA) (tr|A0A0M2EZM6|A0A0M2EZM6_9GAMM) activity. The elongation factor (EF-Tu) and flagellin were also among identified VFs. Twenty-one enzymes important in carbohydrate metabolism were identified. We also identified two F5/8 type C domain-containing proteins, arabinogalactan endo-beta-1,4-galactanase, endoglucanase, and membrane-bound lytic murein transglycosylase each. Other enzymes were peptidoglycan lytic exotransglycylase, glycoside hydrolase, murein transglycosylase B, penicillin-binding protein, endopolygalacturonase, exo-poly-alpha-D-galacturonidase, and an autotransporter protein. The comprehensive antibiotic resistance database (CARD) was used to identify proteins involved in bacterial antimicrobial resistance; hence, such proteins are instrumental in survival. Eight proteins, namely, OmpA, OmpK, LptD, RspA, SecD, FusA, and TolC, were identified. These eight proteins were predicted to facilitate antibiotic efflux, alter the antibiotic target, and reduce permeability to the antibiotic. LptD was among nine out of 72 proteins we identified with a probability >0.9 to be T6SEs. A phospholipase effector and the avirulence protein, suspected to induce HR in plant hosts, were also identified. Other proteins included porins, a phage tail protein, a YjbH domain-containing protein, and TonB dependent plug domain-containing protein.

### 3.6. Contribution of Pbr1692 OMVs to Virulence and Hypersensitive Response

**Figure 7:**
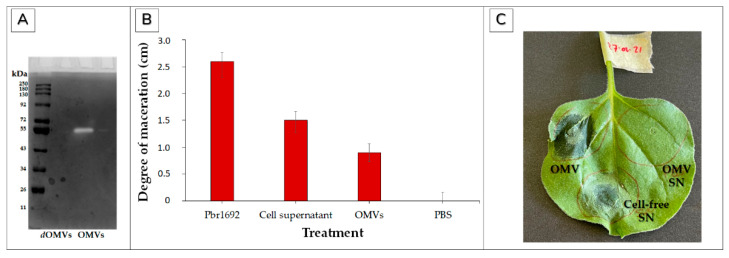
Virulence activities of Pbr1692 OMVs. (**A**) OMV protease activity. A 12% SDS PAGE zymogram gel shows Pbr1692 OMV digestion of gelatine via protease activity. Gelatinase digestion by the OMVs is demonstrated by the white band clearing zone corresponding to a ~55 kDa protein by visualization using Coomassie Brilliant blue staining. Denatured OMVs were used as a negative control. M = marker; dOMVs = denatured OMVs. (**B**) Soft rot of potato tubers by Pbr1692 OMVs. The figure shows OMV-associated maceration of potato tuber tissue three days post-inoculation. The cell-free supernatant was used as the positive control, while PBS was used as a negative control. The results show the means of two independent experiments. Error bars represent the standard deviations from the data. Differences between the effect of the control and OMVs were determined to be statistically significant with *p* < 0.01. (**C**) OMVs elicited a hypersensitive response (HR) in 2-week-old seedlings of *N. benthamiana* 24 hpi. The OMV wash step SN and cell-free supernatant were used as controls.

Using gelatine as the protease target substrate, zymography validated the protease activity of OMVs. In this regard, OMVs displayed a ~55KDa protein demonstrating gelatine hydrolysis of the gelatine incorporated in the gel shown by the white zone clearing at this site (Figure 7A). On the contrary, no activity was observed for denatured/heat-inactivated OMVs (negative control) (Figure 7). Further, OMV cargo macerated tissue of susceptible *Solanum tuberosum* L. cv Mondial three days post-inoculation (Figure 7B). As expected, the maceration degree was highest when potatoes were inoculated with Pbr1692 cells as a positive control compared to cell-free supernatant or OMV cargo (Figure 7B). OMVs and Pbr1692 supernatant also elicited HR, visible at 24 hpi. On the other hand, no activity was observed for the OMV supernatant obtained after the second OMV washing step, implying that all loosely associated proteins were successfully removed and the OMV cargo specifically inflicts observed phenotypes (Figure 7C).

### 3.7. Antimicrobial Activity of OMVs against Dickeya dadantii

**Figure 8:**
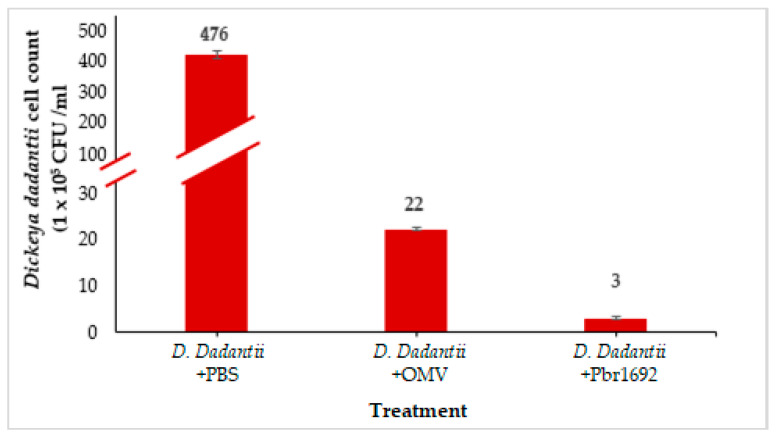
Antimicrobial activity of OMVs. The graph shows *D. dadantii* cells cocultured with PBS as a negative control and its growth inhibition in the presence of Pbr1692 OMVs and Pbr1692 cells. The results show the means of two independent experiments. Error bars represent the standard deviations from the data. Differences between the effect of the PBS-negative control with Pbr1692 cells and OMVs on *D. dadantii* was determined to be statistically significant with *p* < 0.01 in the analysis of variance.

We explored the possibility that the OMVs carry bacterial growth inhibitory components previously shown to give Pbr1692 a competitive advantage against other bacteria [40]. OMVs isolated from the Pbr1692 culture exhibited antibacterial activity against *D. dadantii*, another soft rot pathogen found within the microbial community during potato infection. OMVs reduced the *D. dadantii* cells by approximately 95% (Figure 8). As expected, the proliferation of *D. dadantii* was highly inhibited by Pbr1692 as a positive control compared to when *D. dadantii* was cocultured with PBS as a negative control.

## 4. Discussion

The secretion systems of GNB deliver proteins to different host cell compartments and into the external milieu to invade and evade the host, alienate competitors, and maximize resource usage [12]. However, only a handful of reports contribute to current knowledge of the role of OMVs, specifically those of phytopathogens, in this regard. Since Pbr1692 is an important phytopathogen of potatoes, we were interested in determining the type of vesicles it produces, the cargo ferried by these vesicles, and the potential roles they may play in the biology of this pathogen. Towards this end, we found that in nutritionally rich media, OMVs naturally emerge from distinct areas of Pbr1692 cells during in vitro growth, suggesting that there are dedicated “hot spots” of membrane blebbing. OMVs do not form spontaneously; hence, “hot spots” may be required to maintain cell viability during vesiculation [51]. Vesicles were also embedded in a biofilm-like matrix and, thus, are possible ‘nucleation’ biofilm centers from which biofilm formation can be centered [41,52,53,54,55]. In our previous studies, we found that part of the strategy used by Pbr1692 to invade potato stem xylem tissue is forming biofilms [56,57]. This study brings new insights into the possible contribution of OMVs towards Pbr1692 biofilm formation. In the current study, we found that Pbr1692 produces OMVs and OIMVs. A similar observation was previously reported for *Pectobacterium betavasculorum* IFB5271 and *Pectobacterium zantedeschiae* 9M (formerly *P. atrosepticum*) [11]. The presence of OIMVs makes it difficult to dismiss cytoplasmic proteins, RNA, and DNA as cell lysis contaminants in vesicle preparations [11,58]. Moreover, OIMV constituents seem sensitive; hence, they are enclosed in a double membrane. OIMV production is, thus, specialized, possibly through circularizing membranes of dead cell minority entrapping cytoplasmic proteins and nucleic acids [58]. In contrast to localized blebbing from “hot spots”, explosive or endolysin-triggered cell lysis is a novel biogenesis mode of EOMVs and OIMVs containing periplasmic and cytoplasmic proteins [11,16,17]. There is also sufficient evidence of moonlighting cytosolic proteins and DNA that function on the bacterial cell surface associated with OMVs [59,60]. Other membrane vesicle types and extensions are nano pods, nanotubes, and nanowires [15,20]. Chain-like OMVs and OMV aggregates were observed in this study. Cell–cell communication bridging is assumed to be the reason for the chain-like phenotype [19].

A total of 129 proteins associated with Pbr1692 OMVs was identified. This number compares fairly to other studies, such as one involving *Xylella fastidiosa*, despite differences in culture growth conditions, OMV sizes, and cargo [41,61,62]. The only other study involving *Pectobacterium* spp vesicles using *P. betavasculorum* IFB5271 and *P. zantedeschiae* 9M identified 62 proteins, nearly half the number of proteins identified here [11]. With regard to cargo, most of the proteins identified were outer membrane (36) and cytoplasmic proteins (36). The latter, in early studies, were considered unexpected OMV cargo since OMVs predominantly carried periplasmic and outer membrane components [63]. Extracellular proteins (21) were also found in relatively high numbers as part of OMV cargo. This is not surprising since Pbr1692 directs its extracellular enzymes into the periplasmic space for delivery via the T2SS into the extracellular environment [64]. Therefore, proteins enriched in the periplasm have a higher probability of being loaded into single-membrane vesicles produced by this bacterium.

Among the top 50 most abundant proteins mapped in this study (Table 2), there was a high number of cell and cell part annotated proteins. These included outer membrane proteins, which are generally essential as barriers against stressful conditions. Therefore, this points to a protective role of OMVs to their cargo and producing organism [27]. Among the OMV proteins, antibiotic resistance and nutrient acquisition-related outer membrane proteins critical for survival through roles such as decreasing porin production to reduce the pathogen’s susceptibility to antibiotics and increased membrane integrity were also represented [11,34,65,66,67].

OMV functions currently include pathogenesis, inter- or intraspecies communication, and survival reviewed in [14,32]. Phytopathogen OMV-associated roles include biofilm formation, modulation of plant immunity, and virulence; hence, they are predicted to be intrinsic to their biology, as reviewed by [25]. For example, *Xanthomonas campestris*, *X. fastidiosa*, and *Pseudomonas syringae* pv. tomato T1 are among GNB that release OMVs that contain various virulence factors [24,68,69,70]. Therefore, we expected that OMVs exhibit significance in the fitness of Pbr1692 within microbial communities, its host adaptation, and particularly in virulence as in the other *Pectobacterium* spp. [11]. The protection conferred by vesicles ensures the long-distance delivery of proteins shielded from proteases in the environment [20]. In this study, several PCWDEs and proteases (Pel, Peh, Cel, Prt) were identified virulence factors in OMVs. Moreover, the presence of PCWDEs in OMVs coincided with the abundant oligogalacturonide specific porin (KdgM). KdgM is required to uptake the cell wall degradation products used as a carbon source for bacterial growth after its maceration by PCWDEs [66]. We validated this finding by showing that OMV cargo macerates potato tuber tissue of a high moisture and low starch content susceptible *Solanum tuberosum* L. cv Mondial three days post-inoculation. The degree of maceration by OMVs compared with Pbr1692 cells and the cell-free supernatant emphasized that OMVs contribute as a secretion system.

The OMV proteome profile generated in this study also shows that OMVs carry highly conserved pathogen-associated molecular patterns (PAMPS) known to interact with plant recognition receptors (PRR) to alert the plant’s innate immune system of an attack [26]. In this regard, two flagella proteins were identified in the proteome of Pbr1692 OMVs [26]. Another PAMP found in the OMV proteome was the Elongation factor Tu (EF-Tu). These factors could have contributed to Pbr1692 OMVs’ ability to elicit an immune response in *N. benthamiana*. Other studies characterizing OMV functional roles in phytopathogenesis have also discovered PAMPs such as EF-Tu and polysaccharide A, indicating that vesicles have a conserved mechanism for delivering immunomodulatory molecules from the pathogen to the host [71]. In addition, an avirulence protein whose canonical secretion system is the T2SS was also implicated in inducing HR. Pbr1692 AvrL (tr|A0A0M2F4E7|A0A0M2F4E7_9GAMM) is a homolog of the virulence protein Svx in *Pectobacterium atrosepticum* and an ortholog of AvrM in *D. dadantii*, and both were previously reported to be upregulated in planta [72,73].

Previously, we showed that Pbr1692 has the ability to inhibit the growth of *Dickeya* spp. and other bacteria in vitro or during the infection of its host [40]. Pbr1692 produces phospholipases and other antimicrobial substances, namely, bacteriocins and carbapenem, to kill competitive bacteria. It is possible that this killing is necessitated due to the high demand for nutrients, including iron. As both *D. dadantii* and Pbr typically share the same niche, the challenge posed by *D. dadantii* to Pbr1692 is that PCWDE regulation is often coupled with iron acquisition; hence, there is competition for the available iron and other nutrients [74]. For this reason, Pbr1692 can eliminate its competitors such as *D. dadantii* through the release of toxins. Towards this end, OMVs have a fitness and survival role in these complex microbial communities [7]. As such, our proteomics data reveal that OMVs produced by Pbr1692 contain the CdiA effector (tr|A0A0M2EZM6|A0A0M2EZM6_9GAMM) that causes contact-dependent growth inhibition (CDI). CdiA toxins bind to specific receptors on target bacteria to deliver C-terminal toxin domains to suppress target cell growth [75]. In addition, an antibacterial T6SS substrate, Phospholipase A1 (tr|A0A0M2F4N0|A0A0M2F4N0_9GAMM), was identified with a probability score of 0.886 among the 72 identified OMV and Type 6 Secreted Effector proteins in this study. Predictably, we showed that OMVs isolated from the Pbr1692 culture had antiproliferative effects on *D. dadantii*. OMVs reduced the *D. dadantii* cells by approximately 95%.

Amid the host–pathogen arms race, antibiotics, hot water, UV light, bacteriophages, and steam treatments are among strategies that have been explored to control *Pectobacterium* spp. [76]. OMV deflect bacteriophage attention and mediate antibiotic resistance [19,77]. We predicted that Pbr1692 OMV cargo OmpA, OmpK, TolC, and LptD facilitate resistance to carbapenem, an antibiotic that Pbr1692 itself produces and charges at target competitor bacteria via the T6SS [40].

## 5. Conclusions

We demonstrated that Pbr1692 formed double- and single-membrane OMVs and defined their roles in infection and survival. OMVs appear to be enhancers of Pbr1692’s secretion systems, as shown by their diversified cargo dedicated to bacterial virulence and overall fitness in their ecological niche. The diversity also raises concerns regarding the strictness of this secretion system’s cargo selection based on its leader sequence-independent secretion. This study has provided additional knowledge about the importance of specific proteins, including some CAZymes, antibiotic agents, and T6SEs selected for non-classical secretion in addition to classical secretion by Pbr1692. This work also serves as a springboard for further investigation of OMV crosstalk with the other secretion systems of GNB, such as the T6SS, in the dedicated delivery of anti-plant host effectors.

## Figures and Tables

**Table 1:** Mass spectrometry and Mascot comparison data of Pbr1692 OMVs.

Sample	Total Spectra	Identified Spectra	Identified Peptides	Identified Proteins
OMV1	40,254	1294	629	241
OMV2	39,260	1474	685	262
OMV3	39,726	2167	1002	364
Total	119,240	4935	2316	451

**Table 2:** The top 50 most abundant OMV proteins of Pbr1692 based on iBAQ.

Rank	Protein ID	Description	Molecular Weight [kDa]	Protein Length [aa]	Unique Peptide Number	Gene Ontology IDs	Final Localization	iBAQ
1	tr|A0A6I6X5L4|A0A6I6X5L4_9GAMM	Uncharacterized protein	52.67	492	7	No information	Unknown	7865.322
2	tr|A0A086EQL9|A0A086EQL9_9GAMM	MipA/OmpV family protein	27.47	249	6	GO:0016021	Outer membrane	6915.651
3	tr|A0A0M2F0M9|A0A0M2F0M9_9GAMM	Phage tail sheath family protein	51.19	475	11	No information	Unknown	6680.566
4	tr|A0A6I6XF41|A0A6I6XF41_9GAMM	Porin	39.82	369	12	GO:0006811 GO:0015288 GO:0009279 GO:0046930	Outer membrane	4079.296
5	tr|A0A3S0ZK54|A0A3S0ZK54_9GAMM	Lipoprotein NlpD	35.59	344	7	No information	Outer membrane	2786.763
6	tr|A0A0M2F4E7|A0A0M2F4E7_9GAMM	Avirulence protein	68.42	621	15	No information	Unknown	2730.042
7	tr|A0A6I6X6T7|A0A6I6X6T7_9GAMM	Serralysin	51.22	476	8	No information	Extracellular	2408.248
8	tr|A0A6I6X997|A0A6I6X997_9GAMM	Porin	27.64	243	4	No information	Outer membrane	2390.189
9	tr|A0A086ESP1|A0A086ESP1_9GAMM	30S ribosomal protein S4	23.44	206	5	GO:0003735 GO:0019843 GO:0006412 GO:0015935	Cytoplasmic	2368.547
10	tr|A0A0M2F5U6|A0A0M2F5U6_9GAMM	Tol-Pal system protein TolB	45.89	430	8	GO:0017038 GO:0042597	Periplasmic	2326,679
11	tr|A0A6I6WLG2|A0A6I6WLG2_9GAMM	Endolytic peptidoglycan transglycosylase RlpA	39.08	372	9	GO:0071555 GO:0016829 GO:0008932 GO:0000270 GO:0042834	Extracellular	2241.485
12	tr|A0A0M2F1K8|A0A0M2F1K8_9GAMM	Membrane protein	18.30	171	4	GO:0016021 GO:0009279	Outer membrane	2232.414
13	tr|A0A0M2F2F9|A0A0M2F2F9_9GAMM	Endoglucanase	54.91	505	5	GO:0005576 GO:0030248 GO:0008810 GO:0030245	Extracellular	2195.773
14	tr|A0A3S0ZW77|A0A3S0ZW77_9GAMM	DNA protection during starvation protein	18.47	167	2	GO:0005737 GO:0016722 GO:0006879 GO:0030261 GO:0008199 GO:0006950 GO:0003677	Cytoplasmic	2130.814
15	tr|A0A6I6X0X5|A0A6I6X0X5_9GAMM	Porin	27.26	238	6	No information	Outer membrane	2073.776
16	tr|A0A6I6WXM4|A0A6I6WXM4_9GAMM	TonB-dependent siderophore receptor	85.60	782	17	GO:0005506 GO:0009279 GO:0004872 GO:0015891	Outer membrane	2064.695
17	tr|A0A6I6X4V7|A0A6I6X4V7_9GAMM	Long-chain fatty acid transport protein	46.74	433	7	No information	Outer membrane	1905.703
18	tr|A0A0M2F6B4|A0A0M2F6B4_9GAMM	Peptidoglycan-associated protein OS	18.52	170	3	GO:0016021 GO:0009279	Outer membrane	1732.263
19	tr|A0A086F0V9|A0A086F0V9_9GAMM	30S ribosomal protein S21	8.49	71	2	GO:0003735 GO:0005840 GO:0016787 GO:0005829 GO:0019843 GO:0000028 GO:0044391 GO:0006412 GO:0022627	Cytoplasmic	1724.587
20	tr|A0A0M2F5C1|A0A0M2F5C1_9GAMM	60 kDa chaperonin	57.03	548	12	GO:0005737 GO:0042026 GO:0051082 GO:0005524	Cytoplasmic	1668.284
21	tr|A0A6I6WLX4|A0A6I6WLX4_9GAMM	F5/8 type C domain-containing protein	72.11	683	8	No information	Extracellular	1577.233
22	tr|A0A0M2F2C0|A0A0M2F2C0_9GAMM	Glycine zipper 2TM domain-containing protein	15.48	155	2	GO:0019867	Outer membrane	1402.157
23	tr|A0A6I6X4G9|A0A6I6X4G9_9GAMM	Vitamin B12 transporter BtuB	68.71	625	11	GO:0015235 GO:0046872 GO:0046930 GO:0006811 GO:0015288 GO:0004872 GO:0009279	Outer membrane	1394.331
24	tr|A0A086EV21|A0A086EV21_9GAMM	Major outer membrane lipoprotein Lpp	8.40	78	2	GO:0009279 GO:0019867	Outer membrane	1370.143
25	tr|A0A086EK57|A0A086EK57_9GAMM	Aspartate ammonia-lyase	52.59	479	2	GO:0006099 GO:0006531 GO:0008797	Cytoplasmic	1357.524
26	tr|A0A086ESI7|A0A086ESI7_9GAMM	Elongation factor Tu	43.22	394	8	GO:0005737 GO:0003746 GO:0005622 GO:0003924 GO:0005525	Cytoplasmic	1357.145
27	tr|A0A6I6X1L4|A0A6I6X1L4_9GAMM	Arabinogalactan endo-beta-1,4-galactanase	56.21	507	4	GO:0015926 GO:0031218 GO:0008152	Cytoplasmic membrane	1346.036
28	tr|A0A086EDQ7|A0A086EDQ7_9GAMM	50S ribosomal protein L18 OS	12.71	117	2	GO:0003735 GO:0019843 GO:0005840 GO:0006412	Cytoplasmic	1340.468
29	tr|A0A0M2EXV1|A0A0M2EXV1_9GAMM	Baseplate protein	20.34	193	3	No information	Unknown	1321.959
30	tr|A0A6I6X2M0|A0A6I6X2M0_9GAMM	Flagellar hook-associated protein 1	59.66	564	5	GO:0044780 GO:0005576 GO:0071973 GO:0009424 GO:0005198	Extracellular	1223.784
31	tr|A0A0M2F0F1|A0A0M2F0F1_9GAMM	Membrane protein	23.01	210	4	GO:0016021 GO:0009279	Outer membrane	1198.896
32	tr|A0A086EVT8|A0A086EVT8_9GAMM	Membrane protein	19.83	190	3	No information	Unknown	1155.032
33	tr|A0A6I6X7Z6|A0A6I6X7Z6_9GAMM	Phage tail protein	71.45	663	8	No information	Unknown	1079.253
34	tr|A0A433N765|A0A433N765_9GAMM	Flagellin	30.05	290	7	GO:0005576 GO:0071973 GO:0009420 GO:0005198	Extracellular	1071.265
35	tr|A0A0M2F3M0|A0A0M2F3M0_9GAMM	Pectate lyase	40.57	374	5	GO:0030570 GO:0016829 GO:0000272 GO:0005576 GO:0045490 GO:0046872	Extracellular	979.6334
36	tr|A0A0M2F635|A0A0M2F635_9GAMM	Membrane protein	50.45	463	7	GO:0005215 GO:0019867	Outer membrane	906.776
37	tr|A0A6I6X7V5|A0A6I6X7V5_9GAMM	TonB-dependent receptor	78.19	701	6	GO:0006810 GO:0009279 GO:0004872	Outer membrane	887.6531
38	tr|A0A433N6R3|A0A433N6R3_9GAMM	Flagellar hook-associated protein 2	50.76	474	9	GO:0007155 GO:0005576 GO:0009424	Extracellular	871.272
39	tr|A0A086EFY4|A0A086EFY4_9GAMM	50S ribosomal protein L17	14.73	130	3	GO:0003735 GO:0005840 GO:0006412	Cytoplasmic	870.2862
40	tr|A0A6I6WY80|A0A6I6WY80_9GAMM	Pectate lyase	40.45	375	5	GO:0016829 GO:0005576 GO:0000272	Extracellular	836.9782
41	tr|A0A086EHI7|A0A086EHI7_9GAMM	Membrane-bound lytic murein transglycosylase	39.88	357	3	GO:0008933 GO:0016998 GO:0000270 GO:0071555 GO:0009279	Unknown	805.2442
42	tr|A0A0M2F7U3|A0A0M2F7U3_9GAMM	Penicillin-binding protein activator LpoA	72.40	672	5	GO:0031241 GO:0030234 GO:0008360 GO:0009252	Unknown	770.3906
43	tr|A0A086ESF5|A0A086ESF5_9GAMM	Pectate lyase	40.21	374	5	GO:0030570 GO:0016829 GO:0000272 GO:0005576 GO:0045490 GO:0046872	Extracellular	746.1676
44	tr|A0A0M2EW43|A0A0M2EW43_9GAMM	Phosphate-binding protein PstS	36.84	346	5	GO:0043190 GO:0035435 GO:0042301	Unknown	743.0872
45	tr|A0A0M2F4N0|A0A0M2F4N0_9GAMM	Phospholipase A1	33.35	290	3	GO:0006629 GO:0008970 GO:0016020 GO:0052739 GO:0102567 GO:0102568 GO:0004623 GO:0052740	Outer membrane	650.1864
46	tr|A0A086EA76|A0A086EA76_9GAMM	Ribose-phosphate pyrophosphokinase	34.34	315	3	GO:0005737 GO:0016301 GO:0009156 GO:0000287 GO:0004749 GO:0009165 GO:0009116 GO:0006015 GO:0005524	Cytoplasmic	648.6135
47	tr|A0A086EWE3|A0A086EWE3_9GAMM	Outer membrane protein assembly factor BamA	89.09	810	5	GO:0051205 GO:0016021 GO:0009279 GO:0043165	Outer membrane	646.6694
48	tr|A0A433N5X5|A0A433N5X5_9GAMM	Putative lipoprotein YajI	20.43	189	2	No information	Cytoplasmic membrane	628.1156
49	tr|A0A086E9U3|A0A086E9U3_9GAMM	LPS-assembly lipoprotein LptE	20.37	184	3	GO:0009279 GO:0043165	Outer membrane	617.4362
50	tr|A0A3S1FKD4|A0A3S1FKD4_9GAMM	Penicillin-binding protein 1B	92.49	826	4	GO:0009252 GO:0008955 GO:0016021 GO:0008658 GO:0071555 GO:0008360 GO:0046677 GO:0009274 GO:0008233	Cytoplasmic membrane	312.0136

## Data Availability

The data presented in this study are openly available in [FigShare] at https://doi.org/10.6084/m9.figshare.16573673.v2.

## Supplementary Materials

The following are available online at https://www.mdpi.com/article/10.3390/microorganisms9091918/s1, Figure S1: Size distribution and concentration of nanoparticles in cell-free supernatants of exponential and stationary phase Pbr1692 cultures; Table S1: Pectobacterium brasiliense 1692 Outer Membrane Vesicle associated proteins.
