# Supplementary figures and images for: Isolation and Characterization of Outer Membrane Vesicles of Pectobacterium brasiliense 1692
Source: Microorganisms. 2021 Sep 9;9(9):1918. doi: 10.3390/microorganisms9091918 (PMC8469291; doi:10.3390/microorganisms9091918)

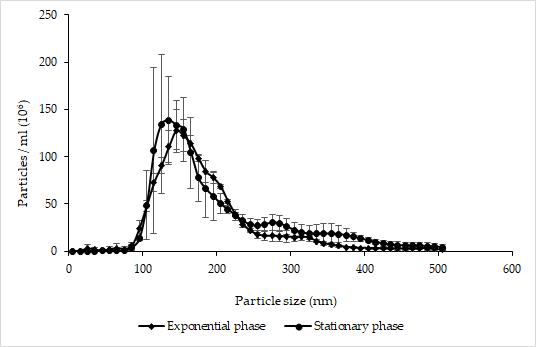

Supplement: Supplementary file 1 [file microorganisms-09-01918-s001.zip › Figure S1.png]
